# Local Treatment of Driveline Infection with Bacteriophages

**DOI:** 10.3390/antibiotics11101310

**Published:** 2022-09-27

**Authors:** Anja Püschel, Romy Skusa, Antonia Bollensdorf, Justus Gross

**Affiliations:** 1 Klinik für Allgemein-, Viszeral-, Gefäß- und Transplantationschirurgie, Universitätsmedizin Rostock, 18057 Rostock, Germany; 2 Institut für Medizinische Mikrobiologie, Virologie und Hygiene, Universitätsmedizin Rostock, 18057 Rostock, Germany

**Keywords:** bacteriophages, phage therapy, application of phages, antibiotic resistance, antibacterial therapy

## Abstract

Drive line infections (DLI) are common infectious complications after left ventricular assist devices (LVAD) implantation. In case of severe or persistent infections, when conservative management fails, the exchange of the total LVAD may become necessary. We present a case of successful treatment of DL infection with a combination of antibiotics, debridement and local bacteriophage treatment.

## 1. Introduction

Left ventricular assist devices (LVAD) are either used as a bridging until heart transplantation or as a final therapy (destination therapy) for advanced heart failure.

With an increase in the number of cases and a longer duration of LVAD support, a new spectrum of long-term complications has opened up. In addition to coagulation disorders, infections are an increasing problem.

Infections occur in 18 to 59% of the cases and may be local, with pocket infection and drive line infection (DLI), or they may be systemic with involvement of the bloodstream and endocarditis. Among them, DLI is the most frequent, with a prevalence of 14–28% [1,2]; it is defined as an infection affecting the soft tissues around the driveline outlet, accompanied by redness, warmness and purulent discharge. Despite it being a confined infection, it has the potential to become systemic with serious consequences.

Medical therapy with broad-spectrum antibiotics is the cornerstone of every treatment. In case of severe infection or ineffectiveness of the primary antibiotic therapy, driveline unroofing and debridement may become necessary.

Reinfections occur frequently and do not seem to be prevented by the use of long-term antibiotics as bacteria embedded in the tissue around the driveline form a surface adherent biofilm leading to a 1000-fold greater tolerance to antibiotics [3,4,5]. Moreover, an increasing occurrence of antibiotic resistance, in particular multi-resistance of Gram-negative germs, poses a challenge for the treatment of implant-associated infections.

The last resource, if severe or persistent infections are present, is the exchange of the total LVAD—an intervention that is associated with high morbidity and mortality [6]. Therefore, less invasive approaches should conservative treatments fail are urgently needed to reduce long-term morbidity and mortality. In this context, bacteriophages and their bacteriolytic activities represent promising therapeutic options.

Bacteriophages are viruses that infect bacteria. They are ubiquitous and highly specific to their bacterial host but are 10 times more numerous, making them the most abundant life forms on Earth, with an estimated 10^31^ bacteriophages on the planet [7]. Already, a decade before the discovery of penicillin, bacteriophages were successfully used to treat bacterial infections. However, at least in the Western world, the initial success was short-lived. The newly emerging antibiotics led to phage treatment being mostly ignored with the exception of some countries of the former Soviet Union and Eastern Europe [8].

Despite longstanding experience in those countries, evidence for the therapeutic application of bacteriophages is scarce and limited to case studies, few preclinical studies and animal models.

## 2. Case Report

In August 2021, a 57-year-old male patient was admitted to the hospital with a local infection on the drive line insertion site. He had no fever, a leukocyte count of 7.65 × 10/l and a serum C reactive protein of 13 mg/L. Blood cultures were negative.

The indication for LVAD implantation in 2018 was a dilative cardiomyopathy and end-stage heart failure. The patient had a history of prior DLI that was treated with antibiotics and surgical debridement. Risk factors for infection were obesity (BMI 36.3 kg/m^2^) and diabetes mellitus. After admission, empiric antibiotic therapy with piperacillin/tazobactam (3 × 4.5 g/day) was initiated. At the site of infection, local wound swabs were taken. The results revealed a mixed driveline infection with *Proteus mirabilis* and *Staphylococcus aureus* that were both sensitive to antibiotic therapy (Table 1).

**Figure 1:**
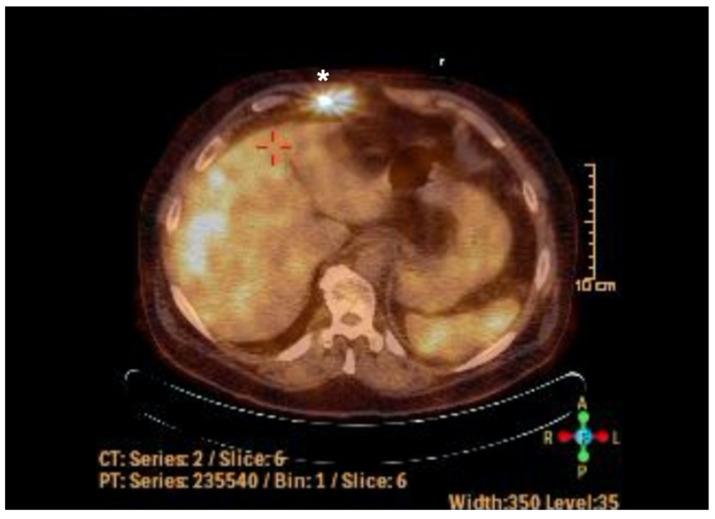
PET-CT scan of the upper abdomen before complex wound treatment. * PET positive driveline of the LVAD-System.

Additionally, the patient underwent a (F18) Fluordeoxyglucose-PET-CT scan 90 min after injection of approximately 220 MBq F-18-FDG on a Sensation 16 Biograph PET/CT scanner (Siemens-Healthineers, Erlangen, Germany). A standardized F18-FDG PET/CT protocol was used including 6 h of fasting, blood glucose levels less than 150 mg/dL, diluted oral contrast (Telebrix, 300 mg; Guerbet, Sulzbach, Germany ) and low-dose CT (26 mAs, 120 kV, 0.5 s per rotation, 5 mm slice thickness) from base of the skull to mid-thigh for attenuation correction. Semiquantitative analysis was performed using a circular region of interest (ROI) (diameter 1.5 cm) with TrueD software (Siemens Medical Solutions, Siemens, Germany) and was normalized for injected dose and patient’s body weight. The scan revealed pathologically increased local metabolic activity of the driveline from the exit point to the entire surrounding subcutaneous adipose tissue up to the abdominal wall muscles (Figure 1). The infection was strictly isolated to the DL exit site without expansion to the pump.

Since the patient had already been unsuccessfully treated surgically for a driveline infection, further local and systemic expansion was to be prevented as a chronic infection is a contraindication to a possible heart transplantation. Therefore, we decided to use an experimental approach with local bacteriophage therapy in addition to renewed surgical therapy.

Local bacteriophage application was planned according to Article 37 of the Declaration of Helsinki (to treat an individual patient for which there are no proven interventions or other known interventions are ineffective, the physician may use an unproven intervention with the patient‘s informed consent) in accordance with our ethics committee (A 2021-0132).

**Figure 2:**
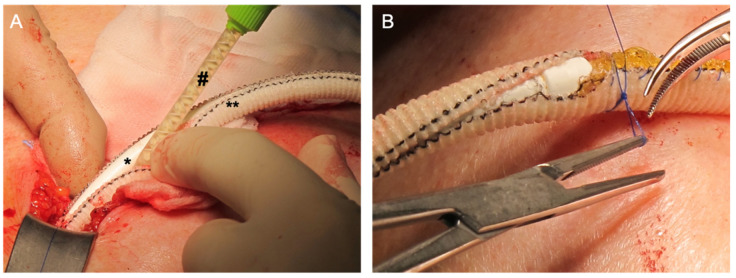
Intraoperative setup: (**A)** the viscous bacteriophage-rich galenic (#) was applied between the driveline (*) and the Gore^®^ Synecore (**). (**B)** The Gore^®^ Synecore was secured with sutures around the driveline before closing the wound.

After obtaining further local swab specimens for microbiological analysis, local debridement of infected tissue, jet lavage with antiseptic (Lavanox, Serag-Wiesner, Naila, Germany) and driveline coating with Gore^®^ Synecore were performed. For this purpose, the Synecore was cut into shape and put around the driveline. Then 20 mL SniPha 360 (1 × 10^7^ CFU) (SniPha 360, Sanubiom GmbH, Fritzens, Austria, Phage 24.com) was diluted in saline and polysaccharide (Starsil, Hemostat Manufacturing GmbH, Velen Germany). The resulting viscous phage-containing fluid was applied between the driveline and the Synecore coating that was further secured by sutures (Figure 2). Additionally, a part of the bacteriophage galenic was applied to the subcutaneous tissue surrounding the driveline before the wound was closed.

SniPha 360 is a commercially available cocktail of lytic bacteriophages against *Escherichia coli*, *Staphylococcus aureus*, *Pseudomonas aeruginosa*, *Streptococcus pyogenes*, *Proteus vulgaris* and *Proteus mirabilis*.

Microbiological analysis of intraoperative samples confirmed infection with *Staphylococcus aureus* and *Proteus mirabilis*.

Susceptibility testing of the patient’s bacterial isolates was performed by spot test. Following an overnight incubation in LB Medium at 37 °C, 200 µL of the bacteria strain suspension was added to the soft agar, mixed and immediately poured on the bottom agar plates. After the bacteria containing top agar solidified, 50 µL of phage suspension was randomly spotted onto the surface of the plates and allowed to dry. The inoculated plates were incubated overnight (18 h) at 37 °C under ambient atmosphere, followed by inspection for lysis zones. A spot test of SniPha 360 on the patient’s strains showed no lysis zone on *S. aureus* and substantial turbidity throughout the cleared lysis zone on *P. mirabilis*.

Because of an uncomplicated postoperative wound healing, we decided against surgical revision and renewal of local phage therapy and continued the conservative treatment. Antibiotic therapy was switched to oral application of cotrimoxazole and the patient was discharged after 20 days with primary wound healing.

Two months later, he was readmitted with a mild local infection at the drive line exit. The microbiological analysis detected only *Staphylococcus aureus*, but not *Proteus mirabilis*. That suggests that the bacteriophages contributed to the treatment success and it is a good example for the correlation of in vitro testing and in vivo results. Long time calculated antibiotic therapy with flucloxacillin was initiated and the patient was discharged after 14 days.

In a follow-up examination 8 months later, no sign of a local or systemic infection was found.

## 3. Discussion

The present case demonstrates the multimodal treatment of an LVAD driveline infection with a combination of antibiotics, surgical debridement and local bacteriophage treatment.

DLI are common infectious complications after LVAD implantation. Besides host risk factors like immunosuppression, diabetes mellitus and obesity, other factors for recurrent or chronic infections are the duration of implants, poor tissue penetration by antibiotics, poor vascular supply, multidrug-resistant microbes and biofilm formation.

Biofilms associated with implanted medical devices represent a significant clinical problem, and often, the total removal of these implants is the only therapeutic option. In the case of LVAD infections, however, this is associated with high morbidity and mortality [6].

As an alternative, less invasive approach, we used a local bacteriophage application.

Bacteriophages, as a non-antibiotic technique for treating bacterial infections, have recently gained popularity. They have been used successfully in humans and other animal species [9,10]. Compared to antibiotics, bacteriophages have a completely different antibacterial mechanism. Phages cannot infect mammalian cells, have no cytotoxic effects on vascular cells and are highly specific to their respective bacterial hosts, thereby protecting the physiological host flora and reducing the risk of secondary infections [11,12].

Furthermore, bacteriophages have been shown to penetrate poorly vascularized tissues and to cross the blood–brain barrier [13]. Many Gram-negative and Gram-positive bacterial infections have been effectively treated in this manner either by local or systemic application [14,15]. Aslam et al. described systemic phage therapy for the first time as an adjuvant to antibiotics to treat left ventricular assist device infection. Since then, only a few case reports or small case studies have been published on the subject of DLI [9,16]. Additionally, the use of specialized and individualized phage mixtures has shown to be an alternative in the fight against multi-drug resistant bacteria as well as in persistent transplant or implant-related infections [11,16,17]. Moreover, in vitro studies have demonstrated that bacteriophages are able to disrupt certain biofilm matrices by exopolysaccharide degradation, bacterial cell infection and subsequent cell lysis [18]. Due to the increasing emergence of biofilm-associated infections such as DLI, there is a need for a therapeutic alternative to antibiotics that could be satisfied by phage therapy [19].

Despite these promising results, bacteriophage treatment is still not common and not officially recommended in the western hemisphere. There is a lack of real clinical trials–the known treatments rely mostly on case reports or small case studies (summarized in Plumet et al. and Aslam et al. [17,20]).

In the present case report, a recurrent local infection with biofilm-forming bacteria was treated with local bacteriophage application after unsuccessful surgical and antibiotic therapy.

Our results are somewhat mixed. Despite the broad host range of the bacteriophage cocktail we used, further bacteriophage in vitro testing revealed no lytic activity on the patient’s *Staphylococcus aureus* strain. This correlated with our clinical observations of reinfection.

As bacteria have co-evolved over the last 3–4 billion years with phages, they developed a variety of mechanisms for preventing viral infections [21]. But with a wide phenotypic variability among phages, even closely related ones, Phage resistant bacteria remain susceptible to other phages of a similar target range [12,22]. Mixing several bacteriophages also helps to minimize the likelihood of bacteria acquiring resistance as well as synergistic antibiotic treatment or higher initial doses [12].

In the present case, however, using a commercially available phage cocktail, the exact composition of the phages and their concentration remain unknown to us. Since primary uncomplicated wound healing occurred, we decided against surgical revision and renewal of local phage therapy and continued the conservative treatment with empiric broad-spectrum antibiotics.

Antibiotics and bacteriophages can have additive or synergistic effects, as demonstrated in vitro and in vivo [22,23].

## 4. Conclusions

In summary, this case report describes the multimodal treatment of a driveline infection. The microbiological data suggest that bacteriophages contributed considerably to the treatment success, and it is also a good example of the correlation of in vitro bacteriophage testing and in vivo results. This case further demonstrates that “one fits all” bacteriophage cocktails, although readily available for immediate clinical use, do not render preceding in vitro testing indispensable.

Many questions, however, remain about the potential of bacteriophage therapy. Further studies are needed to prove and optimize safety, to focus on pharmacokinetics and pharmacodynamics and modes of application as well as on bacteriophage resistance and immune response.

## Figures and Tables

**Table 1:** Susceptibility profile of *Proteus mirabilis* and *Staphylococcus aureus* (S: sensitive, I: intermediate resistant, R: resistant).

Antibiotics	*Proteus mirabilis*	*Staphylococcus aureus*
Oxacillin	R	S
Ampicillin	S	S
Ampicillin/Sulbactam	S	S
Piperacillin/Tazobactam	S	S
Cefuroxime	I	S
Cefotaxime	S	S
Ceftazidime	S	S
Imipenem	I	S
Meropenem	S	S
Gentamicin	S	S
Tetracycline	R	S
Cotimoxazole	S	S
Erythromycin	R	S
Clindamycin	R	S
Vancomycin	R	S
Fosfomycin	S	S
Fusidic acid	R	S
Rifampin	R	S
Linezolid	R	S
Daptomycin	R	S
Tigecycline	R	S
